# Supplementary material for: Completeness of obstetric referral letters/notes from subdistrict to district level in three rural districts in Greater Accra region of Ghana: an implementation research using mixed methods
Source: BMJ Open. 2019 Sep 13;9(9):e029785. doi: 10.1136/bmjopen-2019-029785 (PMC6747881; doi:10.1136/bmjopen-2019-029785)
Supplement: Author's manuscript [file bmjopen-2019-029785.draft_revisions.pdf]

BMJ Open is committed to open peer review. As part of this commitment we make the peer review history of every article we publish publicly available.

When an article is published we post the peer reviewers' comments and the authors' responses online. We also post the versions of the paper that were used during peer review. These are the versions that the peer review comments apply to.

The versions of the paper that follow are the versions that were submitted during the peer review process. They are not the versions of record or the final published versions. They should not be cited or distributed as the published version of this manuscript.

BMJ Open is an open access journal and the full, final, typeset and author-corrected version of record of the manuscript is available on our site with no access controls, subscription charges or pay-per-view fees (http://bmjopen.bmj.com).

If you have any questions on BMJ Open's open peer review process please email info.bmjopen@bmj.com

BMJ Open

Completeness of obstetric referral letters/ notes from sub-district to district level in three rural districts in Greater Accra region of Ghana: an implementation research

Journal:	*BMJ Open*
Manuscript ID	bmjopen-2019-029785
Article Type:	Research
Date Submitted by the Author:	12-Feb-2019
Complete List of Authors:	AMOAKOH-COLEMAN, MARY; Noguchi Memorial Institute for Medical Research, University of Ghana, Epidemiology; University Medical Center, Julius Global Health, Julius Center for Health Sciences and Primary Care<br>Ansah, Evelyn; University of Health and Allied Sciences, Center for Malaria Research<br>Klipstein-Grobusch, Kerstin; University Medical Center, Julius Center for Health Sciences and Primary Care; School of Public Health, University of Witwatersrand, Epidemiology and Biostatistics<br>Arhinful, Daniel; University of Ghana, Epidemiology
Keywords:	OBSTETRICS, referrals, availability, completeness, referral letters or notes

SCHOLARONE™
Manuscripts

Completeness of obstetric referral letters/ notes from sub-district to district level in three rural districts in Greater Accra region of Ghana: an implementation research

Mary Amoakoh-Coleman[1,2], Evelyn K. Ansah[3], Kerstin Klipstein-Grobusch[2,4], Daniel K. Arhinful[1]

[1]Department of Epidemiology, Noguchi Memorial Institute for Medical Research, University of Ghana, Legon, Ghana

[2]Julius Global Health, Julius Center for Health Sciences and Primary Care, University Medical Center Utrecht, Utrecht University, The Netherlands

[3]Center for Malaria Research, Institute of Health Research, University of Health & Allied Sciences, Ho, Ghana

[4]Division of Epidemiology and Biostatistics, School of Public Health, Faculty of Health Sciences, University of the Witwatersrand, Johannesburg, South Africa

Correspondence*

***Email: mcoleman@noguchi.ug.edu.gh; menba19@yahoo.com (MAC)**

k.klipstein-grobusch@umcutrecht.nl (KKG)

eansah@uhas.edu.gh (EKA)

darhinful@noguchi.ug.edu.gh (DKA)

Word Count: 4,940

For peer review only

Abstract

Objective: To assess the completeness of obstetric referral letters/ notes at the district level of healthcare.

Design: An implementation research within three districts of the Greater Accra region using mixed methods approach. At baseline and during the intervention phases, the referral processes for all obstetric referrals from primary level facilities to district hospitals, were documented including, indications for referrals, availability and completeness of referral notes/ forms and how these compare to the national referral policy guidelines. An assessment of before and after intervention availability and completeness of referral forms was carried out.

Setting: Three (3) districts in the Greater Accra region of Ghana.

Participants: All obstetric referrals to and seen at the three district hospitals during the study period (9 months).

Intervention: An enhanced inter-facility referral communication system designed based on existing communication system and consisting of training, provision of communication tools for facilities, formation of facility level referral teams and strengthening feedback mechanisms between facilities.

Outcome: Completeness of obstetric referral letters/ notes.

Results: Proportion of obstetric referrals receiving referral notes improved from 27.2% to 44.3% from the baseline to intervention period. For these notes, most (73.6%) were written using the standard GHS referral forms. Mean completeness (95% CI) of all forms was 71.3% (64.1% – 78.5%) for the study period, improving from 70.7% (60.4% – 80.9%) to 71.9% (61.1% - 82.7%) from baseline to intervention periods. Health workers reported that don't always provide referral notes and that most referral notes are not completely filled due to various reasons.

Conclusions: Referral notes were not provided for most obstetric referrals. The few notes provided were not all completely filled. Interventions such as training of health workers, regular review of referral processes and use of electronic records can help improve both the provision of and completeness of the referral notes.

Keywords: Obstetric, referrals, availability, completeness, referral letters or notes,

For peer review only

Article Summary

Strengths and limitations of this study

- Use of both quantitative and qualitative methods allowed us to both triangulate and explain the findings from the perspective of the health worker who refers patients and writes the letters/ notes.
- Assessment of referral letters/ notes at the referral hospital only (outside the referring facility) did not allow us to assess referring provider and contextual factors associated with the completeness of the referral notes.
- Available resources allowed us to implement and evaluate the intervention package only for a relatively short period, with possibility of limiting the impact of the intervention.

Introduction

The unpredictability and urgency of most obstetric complications and emergencies that require referrals demands that delays are avoided. Maternal referrals are unavoidable due to unequitable distribution of health care resources. Support systems like effective communication are important during obstetric referrals, as they facilitate the needed emergency care process and reduce barriers of distance and time (1). Also, the quality of care for referred patients and referral feedback mechanisms are enhanced when there is an initial direct contact between the referring and receiving physicians (2;3).

Communicating patient information at the time of referral is important for high-quality care and outcome, and care givers at higher levels of care value this information exchange for shared patients (4). Several problems have been identified regarding effective communication by health providers during referrals. These relate to specifying the main reason for and result of consultation, inadequately written medical reports and unclear follow-up plans amongst others (5-9). The absence of this shared information creates dissatisfaction amongst providers of care. The reasons for dissatisfaction include delayed or missing referral letter, missing information in the referral communication, time required to write a referral note, and difficulty in finding a specialist (5;6;8-12). It has been acknowledged that effective communication around referrals facilitates processes needed for referral, including transportation (1). Inter-facility communication makes it possible for the referring facility to confirm that the referral facility has the needed services, provider and logistics for the patient

For peer review only

at the time of referral. The referral center is able to adequately prepare to receive patients when they are informed about the patient ahead of her arrival. This helps to avoid waste of time resulting from referred patients moving from one place to another for the needed care. The referral letter or note serves as a useful communication tool for referrals. However, often inter-facility communication is limited, and written notes offer limited information for patient care because of their quality (13). A review of surgical referrals in a tertiary hospital in Ghana showed incomplete referral forms for all participants, with more missing essential items when structured referral forms are not used compared to when they are used (14).

In Ghana, at the district level of the primary health care, obstetric referrals are from community based planning services (CHPS) compounds and health centres to district hospitals. Upon referral of patients for any condition including obstetric care, a referral note or letter has to be written using a referral form. The filled referral form describes who the patient is, her complaints, general and obstetric examination findings and laboratory investigations, diagnosis, what treatment has been given or started, reason for referral and contact of the referring provider (15). Limited work has been done on quality of obstetric referrals and specifically on the quality of obstetric referral notes in the Ghanaian context (16). Our aim was to assess the completeness of the referral letters/ notes that pregnant women are given when referred from the sub-district level to the district level for care.

Methods

Design & setting: This study is part of an implementation research to evaluate the role of an enhanced inter-facility communication system on the processes and outcomes of maternal referrals in three districts/ municipalities in the Greater Accra region of Ghana from May 2017 to January 2018. It employed a mixed methods approach. The qualitative methods were to enable us interrogate potential explanations behind some of the quantitative findings. Quantitative assessment was undertaken by surveys involving the use of a before and after design while for the qualitative assessment, focus group discussions, non-participant observations and in-depth interviews were conducted. A composite intervention package of an enhanced inter-facility communication system was put in place and run for 4 months after four months of baseline data collection.

The Greater Accra region hosts Ghana’s capital city and has 20 administrative metropolises, municipalities, districts and sub-metropolises. It is mostly urban but has 4 rural districts.

For peer review only

Available resources for this work did not allow us to work in the purely urban districts which have a more complex network of referrals from both public and private facilities The districts we worked in are Ga West, Ada East and Ningo-Prampram districts/ municipalities and are largely rural or semi-urban. Two of the selected districts (Ada East and Ga West) have district hospitals while one (Ningo-Prampram) has a polyclinic as the highest level public facility. It however has a private hospital where patients are referred to, although some patients in this district also get referred to a neighbouring district hospital which is also in another region. The different types of districts with respect to levels of care were used in this study to enable us explore the dynamics in the referral processes for the different types of services available in the district and possible implications for outcomes of care.

The referral form: The Ghana Health Service (GHS) has, as part of its quality control measures in clinical care, designed a standard referral form that describes information that is needed to be passed on to the receiving facility about each referral. This is supplied to all facilities upon request through the medical stores. It comes in duplicate in a booklet, allowing the client to be accompanied with one while the duplicate is kept in the facility for future reference. The referral form (Table 1) is used for referrals for all conditions, including obstetric care, within the GHS, and also during referrals out of the GHS facilities to other facilities in the Ministry of Health (private, quasi-government and tertiary levels). Each woman during antenatal care receives the maternal health record book in which all record concerning the pregnancy, from antenatal through delivery to postnatal care is to be documented. During a referral, a referral form is filled. The variables on the standard form have been presented in Table 1 below.

Table 1: Variables on the referral form to be completed for referred clients

**Health facility information**	**Patient identification**	**Patient clinical information**	**Referring officer identification**
Date	Registration number	Presenting complaints	Name of officer referring
Name and address of referring facility	Name	Examination findings	Position
Name and address of facility referred to	Sex	Temperature	Signature
Time referred	Date of birth	Pulse	Date/ Stamp
Time of departure (if emergency)	Age	Respiratory rate	
	Insurance status	Blood pressure	
	Name and address	Weight	

For peer review only

	of contact person		
	Phone number of contact person	Results of investigations	
		Diagnosis (es)	
		Medical treatment/ management given	
		Reason for referral and comment to next level	

Procedure: Quantitative

A facility audit was conducted for every participating facility in the three districts to ascertain the capacity of the facility to handle referrals with respect to human resources, logistics and supplies, training, protocols and guidelines, referral forms and other related documents. Again, for every participant referred from the primary level facility to the district hospital during the study period, we ascertained whether or not she was given a referral form. For those who had a referral form, the details of the form were captured with respect to the completeness of filling the form. For every variable that was completed on the form, a Yes (1) was assigned and a No (0) when the variable is not completed.

Procedure: Qualitative

Focus group discussions (FGDs) and in-depth interviews (IDIs) were conducted for insight into the processes and outcomes of maternal referrals. There were six focus group discussions at baseline, 2 sets in each district, one for health workers and another for pregnant women and their spouses/ partners/ mothers. There were three sets after the intervention period, one for each district for health workers since they directly benefited from the intervention. Average number of participants per each FGD was 12. District, facility and obstetric/ maternity unit heads provided in-depth interviews at baseline and after the intervention period. Data collected qualitatively included information on indications for referrals, use of referrals notes and completeness of filling them, preparing clients for referrals including giving first aid, the availability and role of inter-facility communication, challenges with referrals, transportation, logistics for referrals, cost of referrals to client, providers and clients' perception about referrals. Discussions and interviews were conducted in English, Ga and Twi.

Intervention

For peer review only

This is an intervention package that ensures suitability and ownership, arrived at through an assessment and understanding of what currently exists and its challenges and how this could be improved pragmatically. The design of the intervention was developed by a team comprising the study co-investigators and a communication expert. The team reviewed and considered existing policy and relevant documents as well as previous and ongoing interventions on the subject of referrals and maternal services from relevant agencies in the Ghana Health service. The final intervention package was guided by what will be feasible and sustainable for the facilities to possibly adopt after this study.

The intervention package consisted of the following activities:

1. Training of health workers on inter-facility referral communication including accurate documentation and use of referral notes.
2. Sharing patient information between referring and referral facilities on all referrals.
3. Provision of communication tools such as working phones and call credits for health workers to facilitate calls and SMS.
4. Designating the task of inter-facility referral communication to someone or a team in the referral facilities (including the specialist in the referral facility), and linking all such agents or teams to all the facilities within a district. These teams had monthly meetings to review maternal referrals.
5. Strengthening and enforcement of feedback mechanisms between referring and referral facilities. This includes monthly SMS reminders to primary level facilities and also onsite visits to primary level facilities to discuss previous referrals and provide feedback by Referral Teams.

Statistical Analysis

Quantitative data was entered into and analysed using IBM SPSS Statistics for Windows, Version 20.0. Armonk, NY: IBM Corp. We estimated the proportions of referred patients who were accompanied with a referral form, those for whom the standard referral forms were used. The percentage completeness for each variable was computed as well as the mean completeness (with 95% confidence interval (CI)) of filling the form. Completeness was further categorized as poor, average or good if the form had less than 50%, between 50-75% and above 75% respectively of the variables on the form completely filled. Comparisons of estimates before and after the intervention were done. Significant differences were estimated

For peer review only

at p = 0.10 due to the relatively short intervention period. Qualitative data was audio recorded, transcribed verbatim and all Twi and Ga responses translated into English. Content analysis was carried out for patterns and emerging themes related to the study objectives.

Ethical approval

The entire study was approved by the Noguchi Memorial Institute for Medical Research (NMIMR) Scientific and Technical Committee (STC) and the Institutional Review Board (NMIMR-IRB CPN 072/16-17) as well as the Ghana Health Service (GHS) Ethical Review Committee (GHS-ERC:11/01/2017). Permission was obtained from the Greater Accra Regional Health Directorate and the participating district health directorates as well as the heads of the selected facilities. Written informed consent, assuring participants' safety, privacy and confidentiality of data provided, was obtained from all participating women and health workers.

Patient and Public Involvement

Patients were indirectly involved in the design of this study. Previous aggregate service data of patients seeking care within the GHS and specifically in the districts involved in this study, informed our design and operationalization of the study.

Also, although the intervention package was proposed before the study, engagement of patients and health workers as part of baseline qualitative data collection informed our modification of and finalization of the intervention.

Recruitment of participants into the study was done by health workers based on the inclusion criteria. District and regional health service workers and managers supported the study.

Results of this study will first be shared with health workers and managers within the study districts as well the Greater Accra region of the GHS, since the intervention focused mainly on health worker practices with respect to obstetric referrals and inter-facility communication.

Secondly since provider practices we studied affect outcome of obstetric care in the three districts, and some of our findings suggest the need to educate women about the usefulness of referrals and thus the need to comply with it, community durbars will be organized in the districts to share relevant findings with the women and relevant stakeholders within the population.

For peer review only

Results

A total of seven hundred and fifty three obstetric referrals were registered in the three districts over the nine month period of the study from twenty-three facilities. The facilities included three hospitals, one polyclinic, eight health centres, eight CHPS compounds and two community clinics. Apart from one hospital and one clinic which were privately owned, the other facilities were government owned. There were 313 referrals during the baseline period and 440 during the invention period. Out of these, only 280 (37.8%) had referral notes. During the baseline period, Ada East, Ga West and Ningo-Prampram had 62, 212 and 39 obstetric referrals respectively, with 30, 38 and 17 referral notes respectively. In the intervention period there were 96, 312 and 32 referrals with 65, 115 and 15 referral notes respectively for the three districts. The specific reason for 210 (75.0%) of obstetric referrals was stated as "for further management". Two hundred and forty seven (88.2%) of the referral notes were written by staff midwives and for eleven (3.9%) notes, the category of the referring health worker was not stated. Figure 1 depicts an improvement in the proportion of clients with referral notes from the 3 districts comparing the baseline and intervention periods.

Figure 1 here: A Graph showing proportion of obstetric referrals with referral notes from the three districts, comparing baseline and intervention periods.

Providing reasons for provision of referral notes in FGDs and IDIs at baseline, some health workers at the primary level facilities reported they always give referral notes to pregnant women before they leave the facility while others stated that they sometimes do not give referral notes, especially if the referral is during the antenatal period and is not for an emergency. However, health workers at the district hospitals reported that not all obstetric referrals come in with referral notes. This trend was similar during the intervention period, but health workers explained that sometimes referred patients refuse to show the referral notes given to them because they want a completely different review and opinion at the referral facility, or they may have gone home and reported to the referral facility much later than expected. A midwife corroborated these points during one of the FGDs in the following statement:

"As she rightly said sometimes when you give them the referral letter alone they throw it away. Most of them don't like it when you refer them to the hospital because they think that they are going to end

For peer review only

up with a caesarean section. So they will throw the referral letter away. So I write a referral letter and I write also in the book [maternal health record book]". (midwife, primary level facility, intervention period FGD, district C)

They also indicated that apart from the referral notes, they have referral notebooks at the facilities in which they keep record of all referrals.

In the FGDs with pregnant women, they reported that although they expect to be given referral notes during referrals, sometimes they are not given one. Some could not tell whether or not they were provided with one because one was not handed over to them with explanation of what it is for. They indicated that the referral noted is useful in sharing their information with the referring facility and that proves that they have indeed been referred.

"…so if they can give us a note to send to the referral destination that is fine ….. otherwise if they have the contact of the facility they should call to tell them about the referred patient……" (Pregnant woman, baseline FGD, District A)

Completeness of referral forms

During the baseline and intervention periods, 47.1% and 85.1% respectively of referral notes were written using the standard GHS referral forms. Other forms used included printed facility adapted versions of the standard form (on which some variables were omitted), health insurance referral forms, and prescription forms (1). In few cases (8) a summary of patient's notes was scribbled in the maternal health record book. For patient identification, 3.0% of notes did not record patient name, 2.0% did not record name of referring facility and 9.0% did not record patient age. There were variations in missing information on the forms for the clinical variables, patient complaints (22.0%), obstetric examination findings (22.0%), blood pressure (4.0%), diagnosis (2.0%) and management given (47.0%). Detailed information on completeness for each variable on the referral forms for baseline, intervention and overall study period is shown in Table 2. Mean completeness of the referral forms (95% CI) for the three districts put together during baseline, intervention and overall period were 70.7% (60.4% – 80.9%), 71.9% (61.1% - 82.7%) and 71.3% (64.1% – 78.5%) respectively. When completeness was re-categorized into poor, average and good, most of the forms (56.7%) were of average completeness (between 50.0% and 75.0% completely filled). There was no

For peer review only

significant association between referring health worker category and category of completeness of the forms. Table 2 shows significant changes in completeness for only a few variables comparing the baseline and intervention periods. Overall, there was no significant change in mean completeness of forms from baseline to intervention period. In terms of category of completeness, there was a significant difference in the performance of the 3 districts *(p=0.01),* but the mean completeness showed no significant difference across the districts as shown in Table 3 where the performance in the three districts are compared.

Table 2: Completeness of obstetric referral notes submitted to the 3 referral hospitals in three districts in the Greater Accra region, comparing baseline and intervention periods

Category	Variable	Entered on Form (N= 85)	Entered on Form (N= 195)	Entered on Form (N= 280)	p-value
		Yes [N (%)] Baseline	Yes [N (%)] Intervention	Yes [N (%)] Overall	
Facility variables	Standard GHS referral form	40 (47.1)	166 (85.1)	206 (73.6)	<0.01
	Name and address of referring facility	81 (95.3)	193 (99.0)	274 (97.9)	0.05
	Patient registration number	26 (30.6)	85 (43.6)	111 (39.6)	0.04
	Time referred	73 (85.9)	181 (92.8)	254 (90.7)	0.07
	Time patient left facility	11 (12.9)	25 (12.8)	36 (12.9)	0.98
Patient Identification	Patient name	82 (96.5)	190 (97.4)	272 (97.1)	0.66
	Age	76 (89.4)	179 (91.8)	255 (91.1)	0.52
	Patient insurance status	54 (63.5)	148 (75.9)	202 (72.1)	0.03
Clinical variables	Patient complaints	62 (72.9)	155 (79.5)	217 (77.5)	0.23
	Obstetric examination findings	64 (75.3)	155 (79.5)	219 (78.2)	0.43
	Blood pressure	83 (97.6)	185 (94.9)	268 (95.7)	0.29
	Weight	47 (55.3)	131 (67.2)	178 (63.6)	0.05
	Laboratory findings	37 (43.5)	74 (37.9)	111 (39.6)	0.38
	Diagnosis	83 (97.6)	192 (98.5)	275 (98.2)	0.64
	Management given	55 (64.7)	92 (47.2)	147 (52.5)	<0.01
	Reason for referral	75 (88.2)	176 (90.3)	252 (90.0)	0.83
	Position of referring officer	77 (90.6)	164 (84.4)	240 (85.7)	0.02
	Signature of referring officer	80 (94.1)	188 (96.4)	268 (95.5)	<0.01
	Phone number of referring officer	35 (41.2)	39 (20.0)	74 (26.4)	<0.01
Completeness categorized	Poor	3 (3.5)	1 (0.5)	4 (1.4)	0.14
	Average	46 (54.1)	113 (57.9)	159 (56.7)	
	Good	36 (42.4)	81 (41.5)	117 (41.8)	
	Mean completeness % (95% CI)	70.67 (60.43 – 80.90)	71.87 (61.10 – 82.65)	71.31 (64.14 - 78.48)	0.87

For peer review only

Table 3: Comparison of completeness of referral notes amongst three districts in Greater Accra region

Period	Variable	District A N (%)	District B N (%)	District C N (%)	p-value
Baseline	Total referral notes	**N= 30**	**N= 38**	**N= 17**	<0.01
	Poor completeness	1 (3.3)	1 (2.6)	1 (5.9)	
	Average completeness	9 (30.0)	32 (84.2)	5 (13.2)	
	Good completeness	20 (66.7)	5 (13.2)	11 (64.7)	
Intervention	Total referral notes	**N= 65**	**N= 115**	**N= 15**	
	Poor completeness	1 (1.5)	0 (0.0)	0 (0.0)	
	Average completeness	42 (64.1)	66 (57.4)	5 (33.3)	
	Good completeness	22 (33.8)	49 (43.6)	10 (66.7)	
Overall period	Total referral notes	**N= 95**	**N= 153**	**N= 32**	0.01
	Poor completeness	2 (2.1)	1 (0.7)	1 (3.1)	
	Average completeness	51 (58.7)	98 (64.1)	10 (31.3)	
	Good completeness	42 (44.2)	54 (35.3)	21 (65.6)	
Overall period	Mean completeness (95% CI)	68.99 (58.13 – 79.86)	71.81 (60.50 – 83.12)	77.34 (67.59 – 87.08)	0.20

Exploring the reasons for incomplete referral forms, health workers and managers indicated during the FGDs and IDIs that, for medico-legal reasons the referral forms are very important and need to be filled out completely and accurately, and also serves as a guide to the health worker as to the essential details to share with the receiving facility during a referral. Incompletely filled forms make it difficult to manage the patient as one is not sure what had already been done for the patient, especially with medication. In some cases the patient is able to confirm what treatment has already been administered. They however admitted that sometimes the referral notes are not completely filled, and this is a challenge for continuing care. They explained that they sometimes they do not fill the form completely because the patient's condition is serious and filling the form can be time wasting.

"Sometimes I do not fill it completely because the patient is in critical condition and has to be moved quickly to the next level." ((midwife, primary level facility, intervention period FGD, district B)

They also stated that most of the information is in the patient's maternal health record book so they find filling the referral form a duplication of effort. Another reason they attributed to not filling in some of the variables, like diagnosis, is that sometimes workers at the referral center criticize them for referring patients with some specific diagnosis. This embarrasses them so they rather leave the diagnosis blank. It is also for similar reasons that they indicate reason for referral in most of the notes as "for further management". Below is a midwife's account:

"Please when we refer the case with a referral letter they [health workers at referral center] should not make comments such as 'What is this, this case too you can't manage?' It happened when I accompanied a referred patient. I felt bad though I didn't write the referral letter and wondered if that is what goes on whenever we refer cases to bigger facilities. That practice is not professional and must stop." (midwife, primary level facility, intervention period FGD, district A)

This position They also believe that any missing information on the referral form can be checked from the maternal health record book which the woman has in her possession.

Availability of forms for referral notes

The standard GHS referral forms are procured from the regional medical stores. The facility audit at baseline showed that 19 out of 22 health facilities (86.4%) had the standard referral form booklets in stock. All hospitals, the one polyclinic and 2 clinics had the referral booklets in stock, with 87.6% and 75.0% respectively of health centres and CHPS compounds having them in stock. In the FGDs and IDIs during baseline, health workers reported that when they have stock-outs, they use photocopies of the forms. They fill two forms or use carbon to duplicate the filled form in order to get a second copy to keep at the facility as required. During the intervention period, a lot of facilities reported having run out of them and so used photocopied versions. When shown different or adapted versions of the standard forms which they had used over the study period, some health workers did not know that they were variants of the standard form without some of the required variables. Some reported that sometimes they write a summary of the indication for referral in the maternal health record book because they do not think the patient's condition warrants a referral note, or they had run out of stock.

Discussion

Main findings

For peer review only

Only 37.8% of obstetric referrals from the three districts during the entire study period had referral notes. Provision of referral notes improved from 27.2% to 44.3% respectively from the baseline to the intervention period. For these notes, most (73.6%) were written using the standard GHS referral forms (47.1% and 85.1% respectively during the baseline and intervention period). Completeness of most forms was within the average category with mean completeness of 71.3% (64.1% – 78.5%) for the study period. During the FGDs and IDIs, health workers explained that they mostly write referral notes for emergency referrals and that most referral notes are not completely filled because other related information is in the maternal health record book which the women carry along to any facility.

Strength and limitations

Strength of this study is the fact that we used both quantitative and qualitative methods and this allowed us to both triangulate and explain the findings from the perspective of the health worker who refers patients and writes the notes.

Patients and referral notes were assessed at the referral hospital only, (outside the referring facility). We were thus unable to assess referring provider and contextual factors associated with the completeness of the referral notes. This is a limitation of the study though the FGDs and IDIs helped us to minimize its effect. Another limitation is the fact that resources, including time, allowed us to implement and evaluate the intervention for a relatively short period than we would have desired. Considering the fact that providers provided more referral notes during the intervention period, it is a possibility that overtime with the intervention in place, mean completeness of the notes may have significantly improved as well.

Implications for obstetric outcomes

Specialists who receive timely patient referral information are more likely to provide optimal care compared to those who do not (17). Obstetric complications can be life threatening and referrals of emergency obstetric cases without referral notes can be potentially time wasting for the receiving care provider (18). Health care is a continuum but with no prior information about a referred patient the whole process of clinical management will have to start from scratch and that is undesirable when the patient needs urgent care. Although health workers gave the impression that all emergency patients get a note, this cannot be confirmed by the

For peer review only

available data. The practice of not providing notes for non-emergency ANC referrals should not be encouraged either. Although the maternal health record book contains ANC information for the woman, studies in Accra, Ghana have shown gaps in ANC data both in the aggregate data and individual client record (19;20). Regarding completeness of notes, incomplete information on medication for example is a serious concern. For example, there are implications for a woman with severe pre-eclampsia who has been given a loading dose of MgSO4 before referral but for which no information exits on reaching the referral center. Does the dose get repeated at the referral centre or not? How does the decision taken affect outcome for the patient? While MgSO4 toxicity or overload has grave consequences which can complicate the management of the patient (21), the lack of the loading dose also puts the patient at a high risk of more seizures which worsen outcomes. This dilemma is avoided when the information is adequately provided on the referral form.

Addressing challenges

Referral notes are very important component of the referral process. The desire is to have all referrals going out with a referral note as reported in one study (6). Unfortunately that was not always the case in our study. The use of standard referral forms or templates has been largely documented to improve documentation of important referral information as well as the overall quality of referral process (22). There is therefore the need to continuously promote the use of the standard GHS referral form among providers of care. There were reported stock-outs of the standard referral forms, necessitating photocopying sometimes for use. This perhaps contributed to some referred patients, especially non-emergency and ANC clients, not getting referral notes. The stock-outs should be addressed with proper stock management of the booklets in the facilities. The referral teams that were formed were tasked to facilitate this role and during the intervention period utilization of the standard form increased. Some clinicians however have expressed preference to rather use their own words to write referrals instead of using a standard form (23), but reviews of such practice shows letters with varying gaps (24-26).

Training and supervision with feedback occurring alone or together have been shown as interventions that improve health worker performance especially in lower resource settings (27-32). Specifically, studies have looked at the benefits of training on how to write referral notes (33) and the use of standard templates to improve quality of such notes from different categories of health workers. During the training in our study, health workers were reminded

For peer review only

of the need to use the standard form, and taken through the process of filling them out accurately. This together with feedback from the referral teams monthly review meetings on referrals contributed to the increase in provision of referral notes as well as use of the standard referral forms from 47.1% to 85.1% over the period, as well. Such interventions that provide regular updates and feedback for health workers should be continued and possibly incorporated into routine facility meetings and engagements. Time required for writing referral notes has been discussed as a problem by health workers (9), but an understanding of the purpose a good referral note serves will help providers take up the task in an efficient manner.

It is important to counsel pregnant women during the ANC period on the importance of referrals and the need to pass on referral notes to the receiving facility so that they desist from hiding the notes. Some referred patients do not show up at the referral facility as has been reported in one study (5). The patients expect clear communication and explanation of the diagnosis or indication for referral, treatment options and follow-up requirements during the referral process (9) and where this is lacking they have a challenge complying with instructions for referrals, including passing on referral notes. Referral facilities should also provide feedback to lower level facilities when their patients come without referral notes or incompletely filled referral notes so that these can be rectified in future.

Completeness of referral notes as shown in this study needs improvement. A study that looked at the content of referral notes or letters, although not specific to obstetric referrals, showed that over 90% of both generalists and consultants agreed that statement of the problem, current medication and reason for referral should be in a referral letter (23). However, several studies show that referral notes from general practitioners often lack critical information such as reason for consultations, socio-psychological factors, or plans for follow-up (4;12;24-26). For example, one study found that although referring physicians provided patient background in 98.0% of referrals, they made the purpose of the referral explicit in only 76.0% (12). Other studies showed no or very little information on physical examination and laboratory investigation on the referral letter (34;35).The lack of adequate information has posed a challenge in using referral letters as tools for medical education (26;36), although practitioners agree that referral notes should be used in professional audits (23). Structured referral forms perform better with respect to completeness of information (14). The referral protocol for GHS specifies that all the variables on the form must be filled in. This leaves no

For peer review only

room for the health worker to use his/ her discretion as to what variable to fill in and what not to for each patient. Although one study (33) showed that a letter with formatted content has the potential to enhance the quality of referral letters, other studies (23;37) showed that general practitioners preferred to have referral forms with less required items, and would rather write a summary based on what they consider important for each patient, rather than fill a form with mandatory fields. In that same study in Australia (33), eight items were rated as essential information by a majority of referral letter recipients who are practitioners and these include the diagnosis, clinical findings, test results, treatment options and recommendations, and prognosis. However information pertaining to medical history, drug or social history was considered less essential. Some practitioners believe that the patient's characteristics as well as the circumstances of each case may vary the information that is essential in each referral note (38). An assessment of the perspectives of the practitioners and the managers who designed the referral form as well as those who use them in Ghana will thus be important to appreciate how much of incompleteness is tolerable within the scope of obstetric referrals.

Electronic records have been shown to improve data completeness (19;39-41). Few studies have examined the effects of electronic medical records (EMRs) on care coordination in general or on the referral process in particular (42).Computer access to chart notes was associated with increased communication between referring physicians and specialists, with specialists receiving written or e-mail referral letters more than twice as often as by telephone or other verbal communication (8;17). Benefits of such electronic communication about referrals include the option for asynchronous communication and opportunities for back-and forth interchange and enhanced rapport (43). Electronic notes are of better quality and also very useful and preferred by practitioners, especially if decision support functions are embedded in them (29;33). Employing their use in the Ghanaian context will be beneficial to the health system in general and referrals specifically.

Conclusion

Referral notes were not provided for most obstetric referrals. The few referral notes were not always completely filled. Interventions such as training of health workers and regular review of referral processes can help improve both the provision and completeness of the referral notes. Use of electronic records should also be explored to benefit from its strengths.

For peer review only

Acknowledgement

The authors are grateful to the NMIMR Postdoctoral Implementation Team for their support as well as the managers and workers of the Ghana Health Service for supporting the process of implementing the intervention package and data collection. They are particularly grateful to Prof. Kwadwo Koram for his guidance through the process.

Funding

This study was funded by the WHO/TDR Postdoctoral grant number B40347 to the NMIMR.

Conflict of interest

Authors declare that they have no competing interest.

Author Contributions

MAC conceived, designed and performed the study, analyzed the data and wrote the paper. EKA contributed to the design of the study and reviewed and approved the final version of the paper. KKG contributed to the design of the study and reviewed and approved the final version of the paper. DKA contributed to the design of the study, analyzed the data, reviewed and approved the final version of the paper.

Data Statement

Data set for this paper is part of a bigger data set from the big study conducted and is currently stored on internal storage systems of NMIMR. We are able to provide data specific to this paper on request, once the purpose for the request fits into the ethics approval we received for the work. Request for the data set specific to this paper may be made to the NMIMR through the corresponding author. Authors will still be working on the bigger data set to answer other questions and objectives of the bigger study so are unable to make it available to others as at now.

Reference List

For peer review only

(1) Murray SF, Pearson SC. Maternity referral systems in developing countries: Current knowledge and future research needs. Social Science & Medicine 2006 May;62(9):2205-15.

(2) Adam Haliq. Referral processes and services for expectant mothers and newborns at the Shai-Osudoku District Hospital 2015.

(3) Africa Initiatives. Addressing Obstetric and Neonatal Complications in Africa from Community and Facility Perspectives, Descriptive Reports from Ghana, Malawi and Uganda. MotherCare/JSI; 1998 Apr 4.

(4) Williams PT, Peet G. Differences in the value of clinical information: referring physicians versus consulting specialists. J Am Board Fam Pract 1994 Jul;7(4):292-302.

(5) Byrd JC, Moskowitz MA. Outpatient consultation: interaction between the general internist and the specialist. J Gen Intern Med 1987 Mar;2(2):93-8.

(6) Cummins RO, Smith RW, Inui TS. Communication Failure in Primary Care: Failure of Consultants to Provide Follow-up Information. JAMA 1980 Feb 3;243(16):1650-2.

(7) Forrest CB, Nutting PA, von Schrader S, Rohde C, Starfield B. Primary Care Physician Specialty Referral Decision Making: Patient, Physician, and Health Care System Determinants. Med Decis Making 2006 Jan 1;26(1):76-85.

(8) Gandhi TK, Sittig DF, Franklin M, Sussman AJ, Fairchild DG, Bates DW. Communication Breakdown in the Outpatient Referral Process. J Gen Intern Med 2000 Sep;15(9):626-31.

(9) Piterman L, Koritsas S. Part II. General practitioner–specialist referral process. Internal Medicine Journal 2005 Jul 26;35(8):491-6.

(10) Bourguet C, Gilchrist V, McCord G. The consultation and referral process. A report from NEON. Northeastern Ohio Network Research Group. J Fam Pract 1998 Jan;46(1):47-53.

(11) Lee T, Pappius EM, Goldman L. Impact of inter-physician communication on the effectiveness of medical consultations. The American Journal of Medicine 1983;74(1):106-12.

(12) McPhee SJ, Lo B, Saika GY, Meltzer R. How good is communication between primary care physicians and subspecialty consultants. Arch Intern Med 1984 Apr 16;144:1265-8.

(13) Harrison R, Clayton W, Wallace P. Can telemedicine be used to improve communication between primary and secondary care? BMJ 1996 Nov 30;313(7069):1377.

(14) Gyedu A, Baah EG, Boakye G, Ohene-Yeboah M, Otupiri E, Stewart BT. Quality of referrals for elective surgery at a tertiary care hospital in a developing country: An

For peer review only

opportunity for improving timely access to and cost-effectiveness of surgical care. International Journal of Surgery 2015;15:74-8.

(15) GHS ICDGHS. Referral Policies and Guidelines. 2007 Mar.

(16) Ngemenenso S.S. An Evaluation of Maternal Referrals in the Sissala East District. Accra: SPH, University of Ghana; 2019.

(17) Stille CJ, McLaughlin TJ, Primack WA, Mazor KM, Wasserman RC. Determinants and Impact of Generalist:Specialist Communication About Pediatric Outpatient Referrals. Pediatrics 2006 Oct 1;118(4):1341.

(18) Martinussen PE. Referral quality and the cooperation between hospital physicians and general practice: The role of physician and primary care factors. Scand J Public Health 2013 Aug 19;41(8):874-82.

(19) Amoakoh-Coleman M, Kayode GA, Brown-Davies C, Agyepong IA, Grobbee DE, Klipstein-Grobusch K, et al. Completeness and accuracy of data transfer of routine maternal health services data in the greater Accra region. BMC Res Notes 2015 Apr 1;8:114.

(20) Amoakoh-Coleman M, Agyepong IA, Zuithoff NPA, Kayode GA, Grobbee DE, Klipstein-Grobusch K, et al. Client Factors Affect Provider Adherence to Clinical Guidelines during First Antenatal Care. PLoS One 2016 Jun 20;11(6):e0157542.

(21) Okusanya BO, Garba KK, Ibrahim HM. The efficacy of 10gram intramuscular loading dose of MgSO(4) in severe preeclampsia/ eclampsia at a tertiary referral centre in Northwest Nigeria. Niger Postgrad Med J 2012 Sep;19(3):143-8.

(22) Tobin-Schnittger P, OΓÇÖDoherty J, OΓÇÖConnor R, OΓÇÖRegan A. Improving quality of referral letters from primary to secondary care: a literature review and discussion paper. 2018;2017/12/07(3):211-22.

(23) Newton J, Eccles M, Hutchinson A. Communication between general practitioners and consultants: what should their letters contain? BMJ 1992 Mar 28;304(6830):821-4.

(24) Butow PN, Dunn SM, Tattersall MH, Jones QJ. Computer-based interaction analysis of the cancer consultation. Br J Cancer 1995 May;71(5):1115-21.

(25) Graham PH. Improving communication with specialists. The case of an oncology clinic. Med J Aust 1994 May 16;160(10):625-7.

(26) Jacobs LG, Pringle MA. Referral letters and replies from orthopaedic departments: opportunities missed. BMJ 1990 Sep 8;301(6750):470-3.

(27) Akbari A, Mayhew A, Al-Alawi MA, Grimshaw J, Winkens R, Glidewell E, et al. Interventions to improve outpatient referrals from primary care to secondary care. Cochrane Database of Systematic Reviews 2008;(4).

For peer review only

(28) Bailey C, Blake C, Schriver M, Cubaka VK, Thomas T, Martin HA. A systematic review of supportive supervision as a strategy to improve primary healthcare services in Sub-Saharan Africa. International Journal of Gynecology & Obstetrics 2015 Nov 6;132(1):117-25.

(29) Corwin P, Bolter T. The effects of audit and feedback and electronic referrals on the quality of primary care referral letters. J Prim Health Care 2014 Dec 1;6(4):324-7.

(30) Davies-Adetugbo AA, Fabiyi AK, Ojoofeitimi EO, Adetugbo K. Breastfeeding training improves health worker performance in rural Nigeria. East Afr Med J 1997 Aug;74(8):510-3.

(31) Naikoba S, Senjovu KD, Mugabe P, McCarthy CF, Riley PL, Kadengye DT, et al. Improved HIV and TB Knowledge and Competence Among Mid-level Providers in a Cluster-Randomized Trial of One-on-One Mentorship for Task Shifting. J Acquir Immune Defic Syndr 2017 Aug 15;75(5):e120-e127.

(32) Nguyen DTK, Leung KK, McIntyre L, Ghali WA, Sauve R. Does Integrated Management of Childhood Illness (IMCI) Training Improve the Skills of Health Workers? A Systematic Review and Meta-Analysis. PLoS One 2013 Jun 12;8(6):e66030.

(33) Tattersall MH, Butow PN, Brown JE, Thompson JF. Improving doctors' letters. Med J Aust 2002 Nov 4;177(9):516-20.

(34) Culshaw D, Clafferty R, Brown K. Let's Get Physical! A Study of General Practitioner's Referral Letters to General Adult Psychiatry ΓÇö Are Physical Examination and Investigation Results Included? Scott Med J 2008 Feb 1;53(1):7-8.

(35) Osinaike BO, Esezobor CI, Akinsola OJ. Quality of Referral Letters to the Paediatric Department of a Tertiary Hospital in Nigeria. Nig Q J Hosp Med 2013 Oct;23(4):273-9.

(36) Gagliardi A. Use of referral reply letters for continuing medical education: a review. J Contin Educ Health Prof 2002;22(4):222-9.

(37) Saha S, Summerwill AJ. Quality of Written Reports Provided by Consultants in Restorative Dentistry to Referring General Dental Practitioners in the West Midlands. Primary Dental Care 13[2], 63-69. 4-1-2006.

Ref Type: Abstract

(38) McConnell D, Butow PN, Tattersall MHN. Improving the letters we write: an exploration of doctor to doctor communication in cancer care. Br J Cancer 1999 May 1;80(3-4):427-37.

(39) Chan KS, Fowles JB, Weiner JP. Review: Electronic Health Records and the Reliability and Validity of Quality Measures: A Review of the Literature. Med Care Res Rev 2010 Feb 11;67(5):503-27.

For peer review only

(40) Hawley G, Jackson C, Hepworth J, Wilkinson SA. Sharing of clinical data in a maternity setting: How do paper hand-held records and electronic health records compare for completeness? BMC Health Serv Res 2014;14(1):650.

(41) Thiru K, Hassey A, Sullivan F. Systematic review of scope and quality of electronic patient record data in primary care. BMJ 2003 May 15;326(7398):1070.

(42) Stille CJ, Jerant A, Bell D, Meltzer D, Elmore JG. Coordinating care across diseases, settings, and clinicians: a key role for the generalist in practice. Ann Intern Med 2005 Apr 19;142(8):700-8.

(43) DO Thomas AH, MD James CB. Telephone reporting in the consultantΓÇôgeneralist relationship. Journal of Evaluation in Clinical Practice 2002 Mar 7;8(1):31-5.

For peer review only

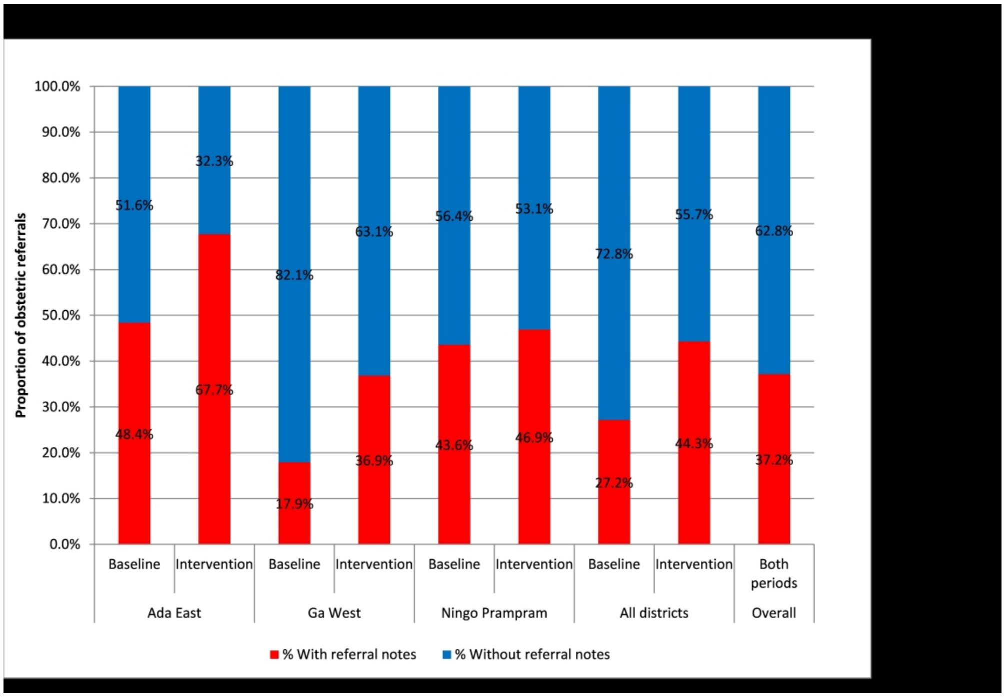

1428x982mm (96 x 96 DPI)

BMJ Open

Completeness of obstetric referral letters/ notes from sub-district to district level in three rural districts in Greater Accra region of Ghana: an implementation research using mixed methods

Journal:	*BMJ Open*
Manuscript ID	bmjopen-2019-029785.R1
Article Type:	Original research
Date Submitted by the Author:	09-Jul-2019
Complete List of Authors:	AMOAKOH-COLEMAN, MARY; University of Ghana Noguchi Memorial Institute for Medical Research, Epidemiology; Utrecht University, Julius Center for Health Sciences and Primary Care, University Medical Center<br>Ansah, Evelyn; University of health and A, Center for Malaria Research<br>Klipstein-Grobusch, Kerstin; Utrecht University, Julius Center for Health Sciences and Primary Care, University Medical Center; Wits University, Biostatistics and Epidemiology, School of Public Health<br>Arhinful, Daniel; University of Ghana Noguchi Memorial Institute for Medical Research, Epidemiology
**Primary Subject Heading**:	Health services research
Secondary Subject Heading:	Epidemiology, Health policy
Keywords:	OBSTETRICS, referrals, availability, completeness, referral letters or notes

SCHOLARONE™ Manuscripts

Completeness of obstetric referral letters/ notes from sub-district to district level in three rural districts in Greater Accra region of Ghana: an implementation research using mixed methods

Mary Amoakoh-Coleman[1,2], Evelyn K. Ansah[3], Kerstin Klipstein-Grobusch[2,4], Daniel K. Arhinful[1]

[1]Department of Epidemiology, Noguchi Memorial Institute for Medical Research, University of Ghana, Legon, Ghana

[2]Julius Global Health, Julius Center for Health Sciences and Primary Care, University Medical Center Utrecht, Utrecht University, The Netherlands

[3]Center for Malaria Research, Institute of Health Research, University of Health & Allied Sciences, Ho, Ghana

[4]Division of Epidemiology and Biostatistics, School of Public Health, Faculty of Health Sciences, University of the Witwatersrand, Johannesburg, South Africa

Correspondence*

***Email: mcoleman@noguchi.ug.edu.gh; menba19@yahoo.com (MAC)**

k.klipstein-grobusch@umcutrecht.nl (KKG)

eansah@uhas.edu.gh (EKA)

darhinful@noguchi.ug.edu.gh (DKA)

Word Count: 5,240

For peer review only

Abstract

Objective: To assess the completeness of obstetric referral letters/ notes at the district level of healthcare.

Design: An implementation research within three districts in Greater Accra region using mixed methods. During baseline and intervention phases, referral processes for all obstetric referrals from lower level facilities seen at the district hospitals were documented including indications for referrals, availability and completeness of referral notes/ forms. An assessment of before and after intervention availability and completeness of referral forms was carried out. Focus group discussions, non-participant observations and in-depth interviews with health workers and pregnant women were conducted for qualitative data.

Setting: Three (3) districts in the Greater Accra region of Ghana.

Participants: Pregnant women referred from lower levels of care to and seen at the district hospital. Health workers within the three districts. Pregnant women attending antenatal clinic in the district and their family members or spouses.

Intervention: An enhanced inter-facility referral communication system consisting of training, provision of communication tools for facilities, formation of hospital referral teams and strengthening feedback mechanisms.

Outcome: Completeness of obstetric referral letters/ notes.

Results: Proportion of obstetric referrals with referral notes improved from 27.2% to 44.3% from the baseline to intervention period., Mean completeness (95% CI) of all forms was 71.3% (64.1% – 78.5%) for the study period, improving from 70.7% (60.4% – 80.9%) to 71.9% (61.1% - 82.7%) from baseline to intervention periods. Health workers reported they don't always provide referral notes and that most referral notes are not completely filled due to various reasons.

Conclusions: Most obstetric referrals did not have referral notes. The few notes provided were not completely filled. Interventions such as training of health workers, regular review of referral processes and use of electronic records can help improve both the provision of and completeness of the referral notes.

Keywords: Obstetric, referrals, availability, completeness, referral letters or notes,

For peer review only

Article Summary

Strengths and limitations of this study

- Use of both quantitative and qualitative methods allowed us to both triangulate and explain the findings from the perspective of the health worker who refers patients and writes the letters/ notes.
- Assessment of referral letters/ notes at the referral hospital only (outside the referring facility) did not allow us to assess referring provider and contextual factors associated with the completeness of the referral notes.
- Also as evident from the results of the qualitative data, it is possible some participants were given referral notes but did not present them at the referral facility.
- Available resources allowed us to implement and evaluate the intervention package only for a relatively short period, with possibility of limiting the impact of the intervention.

Introduction

The unpredictability and urgency of most obstetric complications and emergencies that require referrals demands that delays are avoided. Maternal referrals are unavoidable due to unequitable distribution of health care resources. Support systems like effective communication are important during obstetric referrals, as they facilitate the needed emergency care process and reduce barriers of distance and time (1). Also, the quality of care for referred patients and referral feedback mechanisms are enhanced when there is an initial direct contact between the referring and receiving physicians (2;3).

Communicating patient information at the time of referral is important for high-quality care and outcome, and care givers at higher levels of care value this information exchange for shared patients (4). Several problems have been identified regarding effective communication by health providers during referrals. These relate to specifying the main reason for and result of consultation, inadequately written medical reports and unclear follow-up plans amongst others (5-9). The absence of this shared information creates dissatisfaction amongst providers of care. The reasons for dissatisfaction include delayed or missing referral letter, missing information in the referral communication, time required to write a referral note, and difficulty in finding a specialist (5;6;8-12). It has been acknowledged that effective communication around referrals facilitates processes needed for referral, including

For peer review only

transportation (1). Inter-facility communication makes it possible for the referring facility to confirm that the referral facility has the needed services, provider and logistics for the patient at the time of referral. The referral center is able to adequately prepare to receive patients when they are informed about the patient ahead of her arrival. This helps to avoid waste of time resulting from referred patients moving from one place to another for the needed care. The referral letter or note serves as a useful communication tool for referrals. However, often inter-facility communication is limited, and written notes offer limited information for patient care because of their quality (13). A review of surgical referrals in a tertiary hospital in Ghana showed incomplete referral forms for all participants, with more missing essential items when structured referral forms are not used compared to when they are used (14).

In Ghana, at the district level of the primary health care, obstetric referrals are from lower levels such as community-based health planning and services (CHPS) compounds, community clinics and health centres to the district hospital. Upon referral of patients for any condition including obstetric care, a referral note or letter has to be written using a referral form. The filled referral form describes who the patient is, her complaints, general and obstetric examination findings and laboratory investigations, diagnosis, what treatment has been given or started, reason for referral and contact of the referring provider (15). Limited work has been done on quality of obstetric referrals and specifically on the quality of obstetric referral notes in the Ghanaian context (16). Our aim was to assess the completeness of the referral letters/ notes that pregnant women are given when referred from the sub-district level to the district level for care.

Methods

Design & setting: This study is part of an implementation research to evaluate the role of an enhanced inter-facility communication system on the processes and outcomes of maternal referrals in three districts/ municipalities in the Greater Accra region of Ghana from May 2017 to January 2018. It employed a mixed methods approach. The qualitative methods were to enable us interrogate potential explanations behind some of the quantitative findings. Quantitative assessment was undertaken by surveys involving the use of a before and after design while for the qualitative assessment, focus group discussions, non-participant observations and in-depth interviews were conducted. A composite intervention package of an enhanced inter-facility communication system was put in place and run for 4 months after four months of baseline data collection.

For peer review only

The Greater Accra region hosts Ghana's capital city and has 20 administrative metropolises, municipalities, districts and sub-metropolises. It is mostly urban but has 4 rural districts. Available resources for this work did not allow us to work in the purely urban districts which have a more complex network of referrals from both public and private facilities. The districts we worked in are districts A, B and C (pseudo-names used for anonymity and confidentiality) and are largely rural (2) or peri-urban, with a higher population (1). Two of the selected districts (Districts A and B) have district public hospitals while one (District C) has a polyclinic as the highest-level public facility. It however has a private hospital where patients are referred to, although some patients in this district also get referred to a neighbouring district hospital which is also in another region. The different types of districts with respect to levels of care were used in this study to enable us explore the dynamics in the referral processes for the different types of services available in the district and possible implications for outcomes of care.

The referral form: The Ghana Health Service (GHS) has, as part of its quality control measures in clinical care, designed a standard referral form that describes information that is needed to be passed on to the receiving facility about each referral. This is supplied to all facilities upon request through the medical stores. It comes in duplicate in a booklet, allowing the client to be accompanied with one while the duplicate is kept in the facility for future reference. The referral form (Table 1) is used for referrals for all conditions, including obstetric care, within the GHS, and also during referrals out of the GHS facilities to other facilities in the Ministry of Health (private, quasi-government and tertiary levels). Each woman during antenatal care receives the maternal health record book in which all record concerning the pregnancy, from antenatal through delivery to postnatal care is to be documented. During a referral, a referral form is filled. The variables on the standard form have been presented in Table 1 below.

Table 1: Variables on the referral form to be completed for referred clients

**Health facility information**	**Patient identification**	**Patient clinical information**	**Referring officer identification**
Date	Registration number	Presenting complaints	Name of officer referring
Name and address of referring facility	Name	Examination findings	Position
Name and address of facility referred to	Sex	Temperature	Signature
Time referred	Date of birth	Pulse	Date/ Stamp

For peer review only

Time of departure (if emergency)	Age	Respiratory rate	
	Insurance status	Blood pressure	
	Name and address of contact person	Weight	
	Phone number of contact person	Results of investigations	
		Diagnosis (es)	
		Medical treatment/ management given	
		Reason for referral and comment to next level	

For peer review only

Procedure: Quantitative

A facility audit was conducted for every participating facility in the three districts to ascertain the capacity of the facility to handle referrals with respect to human resources, logistics and supplies, training, protocols and guidelines, referral forms and other related documents. Again, for every participant referred from the primary level facility to and arriving at the district hospital during the study period, we ascertained whether or not she was given a referral form. For those who had a referral form, the details of the form were captured with respect to the completeness of filling the form. For every variable that was completed on the form, a Yes (1) was assigned and a No (0) when the variable is not completed.

Procedure: Qualitative

Focus group discussions (FGDs) and in-depth interviews (IDIs) were conducted for insight into the processes and outcomes of maternal referrals. There were six focus group discussions at baseline, 2 sets in each district, one for health workers and another for pregnant women and their spouses/ partners/ mothers. There were three sets after the intervention period, one for each district for health workers since they directly benefited from the intervention. Average number of participants per each FGD was 12. District, hospital and obstetric/ maternity unit heads and managers provided in-depth interviews at baseline and after the intervention period. Data collected qualitatively included information on indications for referrals, use of referrals notes and completeness of filling them, preparing clients for referrals including giving first aid, the availability and role of inter-facility communication, challenges with referrals, transportation, logistics for referrals, cost of referrals to client, providers and clients' perception about referrals. Weekly non-participant observations to

For peer review only - http://bmjopen.bmj.com/site/about/guidelines.xhtml

describe nature of majority of referrals coming into the three hospitals were done using a checklist. Discussions and interviews were conducted in English, Ga and Twi.

Research assistants were trained on data collections tools and processes. All data collections tools were pretested in a district with similar characteristics before finalized for use.

Intervention

This is an intervention package that ensures suitability and ownership, arrived at through an assessment and understanding of what currently exists and its challenges and how this could be improved pragmatically. The design of the intervention was developed by a team comprising the study co-investigators and a communication expert. The team reviewed and considered existing policy and relevant documents as well as previous and ongoing interventions on the subject of referrals and maternal services from relevant agencies in the Ghana Health service. The final intervention package was guided by what will be feasible and sustainable for the facilities to possibly adopt after this study.

The intervention package consisted of the following activities and Figure 1 shows how this was eventually implemented:

1. Training of health workers on inter-facility referral communication including accurate documentation and use of referral notes.
2. Sharing patient information between referring and referral facilities on all referrals.
3. Provision of communication tools such as working phones and call credits for health workers to facilitate calls and text messaging.
4. Designating the task of inter-facility referral communication to someone or a team in the referral facilities (including the specialist in the referral facility), and linking all such agents or teams to all the facilities within a district. These teams had monthly meetings to review maternal referrals.
5. Strengthening and enforcement of feedback mechanisms between referring and referral facilities. This includes monthly SMS reminders to referring facilities and also onsite visits to these facilities to discuss previous referrals and provide feedback by Referral Teams.

Figure 1 here: Diagrammatic representation of detailed intervention roll-out

Statistical Analysis

For peer review only

Quantitative data was entered into and analysed using IBM SPSS Statistics for Windows, Version 20.0. Armonk, NY: IBM Corp. We estimated the proportions of referred patients who were accompanied with a referral form and those for whom the standard referral forms were used. The percentage completeness for each variable was computed as well as the mean completeness (with 95% confidence interval (CI)) of filling the form. Completeness was further categorized as poor, average or good if the form had less than 50%, between 50-75% and above 75% respectively of the variables on the form completely filled. Comparisons of estimates before and after the intervention were done using chi-squared ($\chi 2$) test. Significant differences were estimated at $p = 0.10$ due to the relatively short intervention period. Qualitative data was audio recorded, transcribed verbatim and all Twi and Ga responses translated into English. Content analysis was carried out by MAC and a research assistant with expertise in qualitative data analysis for patterns and emerging themes related to the study objectives. Differences were resolved through discussion between MAC and DKA. Main themes which were identified and triangulation of the FGDs, non-participant observations and IDIs data, form the basis for reporting on and interpreting study findings.

Ethical approval

The entire study was approved by the Noguchi Memorial Institute for Medical Research (NMIMR) Scientific and Technical Committee (STC) and the Institutional Review Board (NMIMR-IRB CPN 072/16-17) as well as the Ghana Health Service (GHS) Ethical Review Committee (GHS-ERC:11/01/2017). Permission was obtained from the Greater Accra Regional Health Directorate and the participating district health directorates as well as the heads of the selected facilities. Written informed consent, assuring participants' safety, privacy and confidentiality of data provided, was obtained from all participating women and health workers for all parts of the study.

Patient and Public Involvement

Patients were indirectly involved in the design of this study. Previous aggregate service data of patients seeking care within the GHS and specifically in the districts involved in this study, informed our design and operationalization of the study.

Also, although the intervention package was proposed before the study, engagement of patients and health workers as part of baseline qualitative data collection informed our modification of and finalization of the intervention.

For peer review only

Recruitment of participants into the study was done by health workers based on the inclusion criteria. District and regional health service workers and managers supported the study. Results of this study will first be shared with health workers and managers within the study districts as well the Greater Accra region of the GHS, since the intervention focused mainly on health worker practices with respect to obstetric referrals and inter-facility communication.

Secondly since provider practices we studied affect outcome of obstetric care in the three districts, and some of our findings suggest the need to educate women about the usefulness of referrals and thus the need to comply with it, community durbars will be organized in the districts to share relevant findings with the women and relevant stakeholders within the population.

Results

A total of seven hundred and fifty-three obstetric referrals were registered in the three district hospitals over the nine-month period of the study from twenty-three facilities. The facilities included three hospitals, one polyclinic, eight health centres, eight CHPS compounds and two community clinics. Apart from one hospital and one clinic which were privately owned, the other facilities were government owned. There were 313 referrals during the baseline period and 440 during the invention period. Out of these, only 280 (37.8%) had referral notes. During the baseline period, districts A, B and C had 62, 212 and 39 obstetric referrals respectively, with 30, 38 and 17 referral notes respectively. In the intervention period there were 96, 312 and 32 referrals with 65, 115 and 15 referral notes respectively for the three districts. The specific reason for 210 (75.0%) of obstetric referrals was stated as "for further management". Two hundred and forty-seven (88.2%) of the referral notes were written by staff midwives and for eleven (3.9%) notes, the category of the referring health worker was not stated. Figure 2 depicts an improvement in the proportion of clients with referral notes from the 3 districts comparing the baseline and intervention periods.

Figure 2 here: A Graph showing proportion of obstetric referrals with referral notes from the three districts, comparing baseline and intervention periods.

Non participant observations did not show any discrimination in provision of referral note between emergency and non-emergency referrals, but revealed that most emergency referrals

were associated with inter-facility communication about the referral. Providing reasons for provision of referral notes in FGDs and IDIs at baseline, some health workers at the referring facilities reported they always give referral notes to pregnant women before they leave the facility while others stated that they sometimes do not give referral notes, especially if the referral is during the antenatal period and is not for an emergency. However, health workers at the district hospitals reported that not all obstetric referrals come in with referral notes. This trend was similar during the intervention period, but health workers explained that sometimes referred patients refuse to show the referral notes given to them because they want a completely different review and opinion at the referral facility, or they may have gone home and reported to the referral facility much later than expected. A midwife corroborated these points during one of the FGDs in the following statement:

"As she rightly said sometimes when you give them the referral letter alone, they throw it away. Most of them don't like it when you refer them to the hospital because they think that they are going to end up with a caesarean section. So, they will throw the referral letter away. So, I write a referral letter and I write also in the book [maternal health record book]". (midwife, lower level facility, intervention period FGD, district C)

They also indicated that apart from the referral notes, they have referral notebooks at the facilities in which they keep record of all referrals.

In the FGDs with pregnant women, they reported that although they expect to be given referral notes during referrals, sometimes they are not given one. Some could not tell whether or not they were provided with one because one was not handed over to them with explanation of what it is for. They indicated that the referral noted is useful in sharing their information with the referring facility and that proves that they have indeed been referred.

"…so if they can give us a note to send to the referral destination that is fine ….. otherwise if they have the contact of the facility, they should call to tell them about the referred patient……" (Pregnant woman, baseline FGD, District A)

Completeness of referral forms

For peer review only

During the baseline and intervention periods, 47.1% and 85.1% respectively of referral notes were written using the standard GHS referral forms. Other forms used included printed facility adapted versions of the standard form (on which some variables were omitted), health insurance referral forms, and prescription forms (1). In few cases (8) a summary of patient's notes was scribbled in the maternal health record book. For patient identification, 3.0% of notes did not record patient name, 2.0% did not record name of referring facility and 9.0% did not record patient age. There were variations in missing information on the forms for the clinical variables, patient complaints (22.0%), obstetric examination findings (22.0%), blood pressure (4.0%), diagnosis (2.0%) and management given (47.0%). Detailed information on completeness for each variable on the referral forms for baseline, intervention and overall study period is shown in Table 2. Mean completeness of the referral forms (95% CI) for the three districts put together during baseline, intervention and overall period were 70.7% (60.4% – 80.9%), 71.9% (61.1% - 82.7%) and 71.3% (64.1% – 78.5%) respectively. When completeness was re-categorized into poor, average and good, most of the forms (56.7%) were of average completeness (between 50.0% and 75.0% completely filled). There was no significant association between referring health worker category and category of completeness of the forms. Table 2 shows significant changes in completeness for only a few variables comparing the baseline and intervention periods. Overall, there was no significant change in mean completeness of forms from baseline to intervention period. In terms of category of completeness, there was a significant difference in the performance of the 3 districts *(p=0.01),* but the mean completeness showed no significant difference across the districts as shown in Table 3 where the performance in the three districts are compared.

Table 2: Completeness of obstetric referral notes submitted to the 3 referral hospitals in three districts in the Greater Accra region, comparing baseline and intervention periods

Category	Variable	Entered on Form (N= 85)	Entered on Form (N= 195)	Entered on Form (N= 280)	p-value
		Yes [N (%)] Baseline	Yes [N (%)] Intervention	Yes [N (%)] Overall	
Facility variables	Standard GHS referral form	40 (47.1)	166 (85.1)	206 (73.6)	<0.01
	Name and address of referring facility	81 (95.3)	193 (99.0)	274 (97.9)	0.05
	Patient registration number	26 (30.6)	85 (43.6)	111 (39.6)	0.04
	Time referred	73 (85.9)	181 (92.8)	254 (90.7)	0.07
	Time patient left facility	11 (12.9)	25 (12.8)	36 (12.9)	0.98
Patient Identification	Patient name	82 (96.5)	190 (97.4)	272 (97.1)	0.66
	Age	76 (89.4)	179 (91.8)	255 (91.1)	0.52

For peer review only

	Patient insurance status	54 (63.5)	148 (75.9)	202 (72.1)	0.03
Clinical variables	Patient complaints	62 (72.9)	155 (79.5)	217 (77.5)	0.23
	Obstetric examination findings	64 (75.3)	155 (79.5)	219 (78.2)	0.43
	Blood pressure	83 (97.6)	185 (94.9)	268 (95.7)	0.29
	Weight	47 (55.3)	131 (67.2)	178 (63.6)	0.05
	Laboratory findings	37 (43.5)	74 (37.9)	111 (39.6)	0.38
	Diagnosis	83 (97.6)	192 (98.5)	275 (98.2)	0.64
	Management given	55 (64.7)	92 (47.2)	147 (52.5)	<0.01
	Reason for referral	75 (88.2)	176 (90.3)	252 (90.0)	0.83
	Position of referring officer	77 (90.6)	164 (84.4)	240 (85.7)	0.02
	Signature of referring officer	80 (94.1)	188 (96.4)	268 (95.5)	<0.01
	Phone number of referring officer	35 (41.2)	39 (20.0)	74 (26.4)	<0.01
Completeness categorized	Poor	3 (3.5)	1 (0.5)	4 (1.4)	0.14
	Average	46 (54.1)	113 (57.9)	159 (56.7)	
	Good	36 (42.4)	81 (41.5)	117 (41.8)	
	Mean completeness % (95% CI)	70.67 (60.43 – 80.90)	71.87 (61.10 – 82.65)	71.31 (64.14 - 78.48)	0.87

Table 3: Comparison of completeness of referral notes amongst three districts in Greater Accra region

**Period**	**Variable**	**District A N (%)**	**District B N (%)**	**District C N (%)**	**p-value**
Baseline	Total referral notes	**N= 30**	**N= 38**	**N= 17**	<0.01
	Poor completeness	1 (3.3)	1 (2.6)	1 (5.9)	
	Average completeness	9 (30.0)	32 (84.2)	5 (13.2)	
	Good completeness	20 (66.7)	5 (13.2)	11 (64.7)	
Intervention	Total referral notes	**N= 65**	**N= 115**	**N= 15**	
	Poor completeness	1 (1.5)	0 (0.0)	0 (0.0)	
	Average completeness	42 (64.1)	66 (57.4)	5 (33.3)	
	Good completeness	22 (33.8)	49 (43.6)	10 (66.7)	
Overall period	Total referral notes	**N= 95**	**N= 153**	**N= 32**	0.01
	Poor completeness	2 (2.1)	1 (0.7)	1 (3.1)	
	Average completeness	51 (58.7)	98 (64.1)	10 (31.3)	
	Good completeness	42 (44.2)	54 (35.3)	21 (65.6)	
Overall period	Mean completeness (95% CI)	68.99 (58.13 – 79.86)	71.81 (60.50 – 83.12)	77.34 (67.59 – 87.08)	0.20

For peer review only

Exploring the reasons for incomplete referral forms, health workers and managers indicated during the FGDs and IDIs that, for medico-legal reasons the referral forms are very important and need to be filled out completely and accurately, and also serves as a guide to the health worker as to the essential details to share with the receiving facility during a referral. Incompletely filled forms make it difficult to manage the patient as one is not sure what had already been done for the patient, especially with medication. They however admitted that sometimes the referral notes are not completely filled, and this is a challenge for continuing care. They explained that they sometimes they do not fill the form completely because the patient's condition is serious and filling the form can be time wasting.

"Sometimes I do not fill it completely because the patient is in critical condition and has to be moved quickly to the next level." ((midwife, lower level facility, intervention period FGD, district B)

They also stated that most of the information is in the patient's maternal health record book so they find filling the referral form a duplication of effort. Another reason they attributed to not filling in some of the variables, like diagnosis, is that sometimes workers at the referral center criticize them for referring patients with some specific diagnosis. This embarrasses them so they rather leave the diagnosis blank. It is also for similar reasons that they indicate reason for referral in most of the notes as "for further management". Below is a midwife's account:

"Please when we refer the case with a referral letter they [health workers at referral center] should not make comments such as 'What is this, this case too you can't manage?' It happened when I accompanied a referred patient. I felt bad though I didn't write the referral letter and wondered if that is what goes on whenever we refer cases to bigger facilities. That practice is not professional and must stop." (midwife, lower level facility, intervention period FGD, district A)

This position They also believe that any missing information on the referral form can be checked from the maternal health record book which the woman has in her possession.

Availability of forms for referral notes

The standard GHS referral forms are procured from the regional medical stores. The facility audit at baseline showed that 19 out of 22 health facilities (86.4%) had the standard referral form booklets in stock. All hospitals, the one polyclinic and 2 clinics had the referral booklets in stock, with 87.6% and 75.0% respectively of health centres and CHPS compounds having

For peer review only

them in stock. In the FGDs and IDIs during baseline, health workers and their managers reported that when they have stock-outs, they use photocopies of the forms. They fill two forms or use carbon to duplicate the filled form in order to get a second copy to keep at the facility as required. During the intervention period, a lot of facilities reported having run out of them and so used photocopied versions. When shown different or adapted versions of the standard forms which they had used over the study period, some health workers did not know that they were variants of the standard form without some of the required variables. Some reported that sometimes they write a summary of the indication for referral in the maternal health record book because they do not think the patient's condition warrants a referral note, or they had run out of stock.

Discussion

Main findings

Only 37.8% of obstetric referrals from the three districts during the entire study period had referral notes. Provision of referral notes improved from 27.2% to 44.3% respectively from the baseline to the intervention period. For these notes, most (73.6%) were written using the standard GHS referral forms (47.1% and 85.1% respectively during the baseline and intervention period). Completeness of most forms was within the average category with mean completeness of 71.3% (64.1% – 78.5%) for the study period. During the FGDs and IDIs, health workers explained that they mostly write referral notes for emergency referrals and that most referral notes are not completely filled because other related information is in the maternal health record book which the women carry along to any facility.

Strength and limitations

Strength of this study is the fact that we used both quantitative and qualitative methods and this allowed us to both triangulate and explain the findings from the perspective of the health worker who refers patients and writes the notes.

Patients and referral notes were assessed at the referral hospital only, (outside the referring facility). We were thus unable to assess referring provider and contextual factors associated with the completeness of the referral notes. This is a limitation of the study though the FGDs and IDIs helped us to minimize its effect. Another limitation is the fact that resources,

For peer review only

including time, allowed us to implement and evaluate the intervention for a relatively short period than we would have desired. Considering the fact that providers provided more referral notes during the intervention period, it is a possibility that over time, with the intervention in place, mean completeness of the notes may have significantly improved as well.

Implications for obstetric outcomes

Specialists who receive timely patient referral information are more likely to provide optimal care compared to those who do not (17). Obstetric complications can be life threatening and referrals of emergency obstetric cases without referral notes can be potentially time wasting for the receiving care provider (18). Health care is a continuum but with no prior information about a referred patient the whole process of clinical management will have to start from scratch and that is undesirable when the patient needs urgent care. Although health workers gave the impression that all emergency patients get a note, this cannot be confirmed by the available data. The practice of not providing notes for non-emergency antenatal care (ANC)referrals should not be encouraged either. Although the maternal health record book contains ANC information for the woman, studies in Accra, Ghana have shown gaps in ANC data both in the aggregate data and individual client record (19;20). Regarding completeness of notes, incomplete information on medication for example is a serious concern. For example, there are implications for a woman with severe pre-eclampsia who has been given a loading dose of the medicine magnesium sulphate ($MgSO_4$) before referral but for which no information exits on reaching the referral center. Does the dose get repeated at the referral centre or not? How does this decision taken affect outcome for the patient? While $MgSO_4$ toxicity or overload has grave consequences which can complicate the management of the patient (21), the lack of the loading dose also puts the patient at a high risk of more seizures which worsen outcomes. This dilemma is avoided when the information is adequately provided on the referral form.

Addressing challenges

Referral notes are very important component of the referral process. The desire is to have all referrals going out with a referral note as reported in one study (6). Unfortunately, that was not always the case in our study. The use of standard referral forms or templates has been largely documented to improve documentation of important referral information as well as

For peer review only

the overall quality of referral process (22). There is therefore the need to continuously promote the use of the standard GHS referral form among providers of care. There were reported stock-outs of the standard referral forms, necessitating photocopying sometimes for use. This perhaps contributed to some referred patients, especially non-emergency and ANC clients, not getting referral notes. The stock-outs should be addressed with proper stock management of the booklets in the facilities. The referral teams that were formed were tasked to facilitate this role and during the intervention period utilization of the standard form increased. Some clinicians however have expressed preference to rather use their own words to write referrals instead of using a standard form (23), but reviews of such practice shows letters with varying gaps (24-26).

Training and supervision with feedback occurring alone or together have been shown as interventions that improve health worker performance especially in lower resource settings (27-32). Specifically, studies have looked at the benefits of training on how to write referral notes (33) and the use of standard templates to improve quality of such notes from different categories of health workers. During the training in our study, health workers were reminded of the need to use the standard form, and taken through the process of filling them out accurately. This together with feedback from the referral teams monthly review meetings on referrals contributed to the increase in provision of referral notes as well as use of the standard referral forms from 47.1% to 85.1% over the period, as well. Such interventions that provide regular updates and feedback for health workers should be continued and possibly incorporated into routine facility meetings and engagements. Time required for writing referral notes has been discussed as a problem by health workers (9), but an understanding of the purpose a good referral note serves will help providers take up the task in an efficient manner.

It is important to counsel pregnant women during the ANC period on the importance of referrals and the need to pass on referral notes to the receiving facility so that they desist from hiding the notes. Some referred patients do not show up at the referral facility as has been reported in one study (5). The patients expect clear communication and explanation of the diagnosis or indication for referral, treatment options and follow-up requirements during the referral process (9) and where this is lacking they have a challenge complying with instructions for referrals, including passing on referral notes. Referral facilities should also

For peer review only

provide feedback to lower level facilities when their patients come without referral notes or incompletely filled referral notes so that these can be rectified in future.

Completeness of referral notes as shown in this study needs improvement. A study that looked at the content of referral notes or letters, although not specific to obstetric referrals, showed that over 90% of both generalists and consultants agreed that statement of the problem, current medication and reason for referral should be in a referral letter (23). However, several studies show that referral notes from general practitioners often lack critical information such as reason for consultations, socio-psychological factors, or plans for follow-up (4;12;24-26). For example, one study found that although referring physicians provided patient background in 98.0% of referrals, they made the purpose of the referral explicit in only 76.0% (12). Other studies showed no or very little information on physical examination and laboratory investigation on the referral letter (34;35).The lack of adequate information has posed a challenge in using referral letters as tools for medical education (26;36), although practitioners agree that referral notes should be used in professional audits (23). Structured referral forms perform better with respect to completeness of information (14). The referral protocol for GHS specifies that all the variables on the form must be filled in. This leaves no room for the health worker to use his/ her discretion as to what variable to fill in and what not to for each patient. Although one study (33) showed that a letter with formatted content has the potential to enhance the quality of referral letters, other studies (23;37) showed that general practitioners preferred to have referral forms with less required items, and would rather write a summary based on what they consider important for each patient, rather than fill a form with mandatory fields. In that same study in Australia (33), eight items were rated as essential information by a majority of referral letter recipients who are practitioners and these include the diagnosis, clinical findings, test results, treatment options and recommendations, and prognosis. However, information pertaining to medical history, drug or social history was considered less essential. Some practitioners believe that the patient's characteristics as well as the circumstances of each case may vary the information that is essential in each referral note (38). An assessment of the perspectives of the practitioners and the managers who designed the referral form as well as those who use them in Ghana will thus be important to appreciate how much of incompleteness is tolerable within the scope of obstetric referrals.

For peer review only

Electronic records have been shown to improve data completeness (19;39-41). Few studies have examined the effects of electronic medical records (EMRs) on care coordination in general or on the referral process in particular (42).Computer access to chart notes was associated with increased communication between referring physicians and specialists, with specialists receiving written or e-mail referral letters more than twice as often as by telephone or other verbal communication (8;17). Benefits of such electronic communication about referrals include the option for asynchronous communication and opportunities for back-and forth interchange and enhanced rapport (43). Electronic notes are of better quality and also very useful and preferred by practitioners, especially if decision support functions are embedded in them (29;33). Consideration can be given to linking them to essential health system components such as health insurance claims, with mandatory fields that cannot be skipped to optimize completeness of records. In another other resource-rich setting, in the field of neurosurgery though, an online referral system was tested and health workers found it very useful in communication and completing required documentation (44). Employing their use in the Ghanaian context will be beneficial to the health system in general and referrals specifically.

For peer review only

Conclusion

Referral notes were not provided for most obstetric referrals. The few referral notes were not always completely filled. Interventions such as training of health workers and regular review of referral processes can help improve both the provision and completeness of the referral notes. Use of electronic records should also be explored to benefit from its strengths.

Acknowledgement

The authors are grateful to the NMIMR Postdoctoral Implementation Team for their support as well as the managers and workers of the Ghana Health Service for supporting the process of implementing the intervention package and data collection. They are particularly grateful to Prof. Kwadwo Koram for his guidance through the process.

Funding

This study was funded by the WHO/TDR Postdoctoral grant number B40347 to the NMIMR.

Conflict of interest

Authors declare that they have no competing interest.

Author Contributions

MAC conceived, designed and performed the study, analyzed the data and wrote the paper. EKA contributed to the design of the study and reviewed and approved the final version of the paper. KKG contributed to the design of the study and reviewed and approved the final version of the paper. DKA contributed to the design of the study, analyzed the data, reviewed and approved the final version of the paper.

Data Statement

Data set for this paper is part of a bigger data set from the big study conducted and is currently stored on internal storage systems of NMIMR. We are able to provide data specific to this paper on request, once the purpose for the request fits into the ethics approval we received for the work. Request for the data set specific to this paper may be made to the NMIMR through the corresponding author. Authors will still be working on the bigger data set to answer other questions and objectives of the bigger study so are unable to make it available to others as at now.

For peer review only

Reference List

(1) Murray SF, Pearson SC. Maternity referral systems in developing countries: Current knowledge and future research needs. Social Science & Medicine 2006 May;62(9):2205-15.

(2) Adam Haliq. Referral processes and services for expectant mothers and newborns at the Shai-Osudoku District Hospital. Master's Thesis University of Ghana; 2015. http://ugspace.ug.edu.gh

(3) Africa Initiatives. Addressing Obstetric and Neonatal Complications in Africa from Community and Facility Perspectives: Descriptive Reports from Ghana, Malawi and Uganda. A Report published by MotherCare/JSI; 1998 Apr 4.

(4) Williams PT, Peet G. Differences in the value of clinical information: referring physicians versus consulting specialists. J Am Board Fam Pract 1994 Jul;7(4):292-302.

For peer review only - http://bmjopen.bmj.com/site/about/guidelines.xhtml

(5) Byrd JC, Moskowitz MA. Outpatient consultation: interaction between the general internist and the specialist. J Gen Intern Med 1987 Mar;2(2):93-8.

(6) Cummins RO, Smith RW, Inui TS. Communication Failure in Primary Care: Failure of Consultants to Provide Follow-up Information. JAMA 1980 Feb 3;243(16):1650-2.

(7) Forrest CB, Nutting PA, von Schrader S, Rohde C, Starfield B. Primary Care Physician Specialty Referral Decision Making: Patient, Physician, and Health Care System Determinants. Med Decis Making 2006 Jan 1;26(1):76-85.

(8) Gandhi TK, Sittig DF, Franklin M, Sussman AJ, Fairchild DG, Bates DW. Communication Breakdown in the Outpatient Referral Process. J Gen Intern Med 2000 Sep;15(9):626-31.

(9) Piterman L, Koritsas S. Part II. General practitioner–specialist referral process. Internal Medicine Journal 2005 Jul 26;35(8):491-6.

(10) Bourguet C, Gilchrist V, McCord G. The consultation and referral process. A report from NEON. Northeastern Ohio Network Research Group. J Fam Pract 1998 Jan;46(1):47-53.

(11) Lee T, Pappius EM, Goldman L. Impact of inter-physician communication on the effectiveness of medical consultations. The American Journal of Medicine 1983;74(1):106-12.

(12) McPhee SJ, Lo B, Saika GY, Meltzer R. How good is communication between primary care physicians and subspecialty consultants. Arch Intern Med 1984 Apr 16;144:1265-8.

(13) Harrison R, Clayton W, Wallace P. Can telemedicine be used to improve communication between primary and secondary care? BMJ 1996 Nov 30;313(7069):1377.

(14) Gyedu A, Baah EG, Boakye G, Ohene-Yeboah M, Otupiri E, Stewart BT. Quality of referrals for elective surgery at a tertiary care hospital in a developing country: An opportunity for improving timely access to and cost-effectiveness of surgical care. International Journal of Surgery 2015;15:74-8.

(15) GHS ICDGHS. Referral Policies and Guidelines. 2007 Mar.

(16) Ngemenenso S.S. An Evaluation of Maternal Referrals in the Sissala East District [Public Health]. Accra: A Master's Thesis, SPH, University of Ghana; 2019. http://ugspace.ug.edu.gh

(17) Stille CJ, McLaughlin TJ, Primack WA, Mazor KM, Wasserman RC. Determinants and Impact of Generalist:Specialist Communication About Pediatric Outpatient Referrals. Pediatrics 2006 Oct 1;118(4):1341.

For peer review only

(18) Martinussen PE. Referral quality and the cooperation between hospital physicians and general practice: The role of physician and primary care factors. Scand J Public Health 2013 Aug 19;41(8):874-82.

(19) Amoakoh-Coleman M, Kayode GA, Brown-Davies C, Agyepong IA, Grobbee DE, Klipstein-Grobusch K, et al. Completeness and accuracy of data transfer of routine maternal health services data in the greater Accra region. BMC Res Notes 2015 Apr 1;8:114.

(20) Amoakoh-Coleman M, Agyepong IA, Zuithoff NPA, Kayode GA, Grobbee DE, Klipstein-Grobusch K, et al. Client Factors Affect Provider Adherence to Clinical Guidelines during First Antenatal Care. PLoS One 2016 Jun 20;11(6):e0157542.

(21) Okusanya BO, Garba KK, Ibrahim HM. The efficacy of 10gram intramuscular loading dose of MgSO(4) in severe preeclampsia/ eclampsia at a tertiary referral centre in Northwest Nigeria. Niger Postgrad Med J 2012 Sep;19(3):143-8.

(22) Tobin-Schnittger P, OΓÇÖDoherty J, OΓÇÖConnor R, OΓÇÖRegan A. Improving quality of referral letters from primary to secondary care: a literature review and discussion paper. 2018;2017/12/07(3):211-22.

(23) Newton J, Eccles M, Hutchinson A. Communication between general practitioners and consultants: what should their letters contain? BMJ 1992 Mar 28;304(6830):821-4.

(24) Butow PN, Dunn SM, Tattersall MH, Jones QJ. Computer-based interaction analysis of the cancer consultation. Br J Cancer 1995 May;71(5):1115-21.

(25) Graham PH. Improving communication with specialists. The case of an oncology clinic. Med J Aust 1994 May 16;160(10):625-7.

(26) Jacobs LG, Pringle MA. Referral letters and replies from orthopaedic departments: opportunities missed. BMJ 1990 Sep 8;301(6750):470-3.

(27) Akbari A, Mayhew A, Al-Alawi MA, Grimshaw J, Winkens R, Glidewell E, et al. Interventions to improve outpatient referrals from primary care to secondary care. Cochrane Database of Systematic Reviews 2008;(4).

(28) Bailey C, Blake C, Schriver M, Cubaka VK, Thomas T, Martin HA. A systematic review of supportive supervision as a strategy to improve primary healthcare services in Sub-Saharan Africa. International Journal of Gynecology & Obstetrics 2015 Nov 6;132(1):117-25.

(29) Corwin P, Bolter T. The effects of audit and feedback and electronic referrals on the quality of primary care referral letters. J Prim Health Care 2014 Dec 1;6(4):324-7.

(30) Davies-Adetugbo AA, Fabiyi AK, Ojoofeitimi EO, Adetugbo K. Breastfeeding training improves health worker performance in rural Nigeria. East Afr Med J 1997 Aug;74(8):510-3.

(31) Naikoba S, Senjovu KD, Mugabe P, McCarthy CF, Riley PL, Kadengye DT, et al. Improved HIV and TB Knowledge and Competence Among Mid-level Providers in a Cluster-Randomized Trial of One-on-One Mentorship for Task Shifting. J Acquir Immune Defic Syndr 2017 Aug 15;75(5):e120-e127.

(32) Nguyen DTK, Leung KK, McIntyre L, Ghali WA, Sauve R. Does Integrated Management of Childhood Illness (IMCI) Training Improve the Skills of Health Workers? A Systematic Review and Meta-Analysis. PLoS One 2013 Jun 12;8(6):e66030.

(33) Tattersall MH, Butow PN, Brown JE, Thompson JF. Improving doctors' letters. Med J Aust 2002 Nov 4;177(9):516-20.

(34) Culshaw D, Clafferty R, Brown K. Let's Get Physical! A Study of General Practitioner's Referral Letters to General Adult Psychiatry ΓÇö Are Physical Examination and Investigation Results Included? Scott Med J 2008 Feb 1;53(1):7-8.

(35) Osinaike BO, Esezobor CI, Akinsola OJ. Quality of Referral Letters to the Paediatric Department of a Tertiary Hospital in Nigeria. Nig Q J Hosp Med 2013 Oct;23(4):273-9.

(36) Gagliardi A. Use of referral reply letters for continuing medical education: a review. J Contin Educ Health Prof 2002;22(4):222-9.

(37) Saha S, Summerwill AJ. Quality of Written Reports Provided by Consultants in Restorative Dentistry to Referring General Dental Practitioners in the West Midlands. Primary Dental Care 13[2], 63-69. 4-1-2006.

Ref Type: Abstract

(38) McConnell D, Butow PN, Tattersall MHN. Improving the letters we write: an exploration of doctor to doctor communication in cancer care. Br J Cancer 1999 May 1;80(3-4):427-37.

(39) Chan KS, Fowles JB, Weiner JP. Review: Electronic Health Records and the Reliability and Validity of Quality Measures: A Review of the Literature. Med Care Res Rev 2010 Feb 11;67(5):503-27.

(40) Hawley G, Jackson C, Hepworth J, Wilkinson SA. Sharing of clinical data in a maternity setting: How do paper hand-held records and electronic health records compare for completeness? BMC Health Serv Res 2014;14(1):650.

(41) Thiru K, Hassey A, Sullivan F. Systematic review of scope and quality of electronic patient record data in primary care. BMJ 2003 May 15;326(7398):1070.

(42) Stille CJ, Jerant A, Bell D, Meltzer D, Elmore JG. Coordinating care across diseases, settings, and clinicians: a key role for the generalist in practice. Ann Intern Med 2005 Apr 19;142(8):700-8.

For peer review only

(43) DO Thomas AH, MD James CB. Telephone reporting in the consultantΓÇôgeneralist relationship. Journal of Evaluation in Clinical Practice 2002 Mar 7;8(1):31-5.

(44) Amarouche M, Neville JJ, Deacon S, Kalyal N, Adams N, Cheserem B, et al.

For peer review only

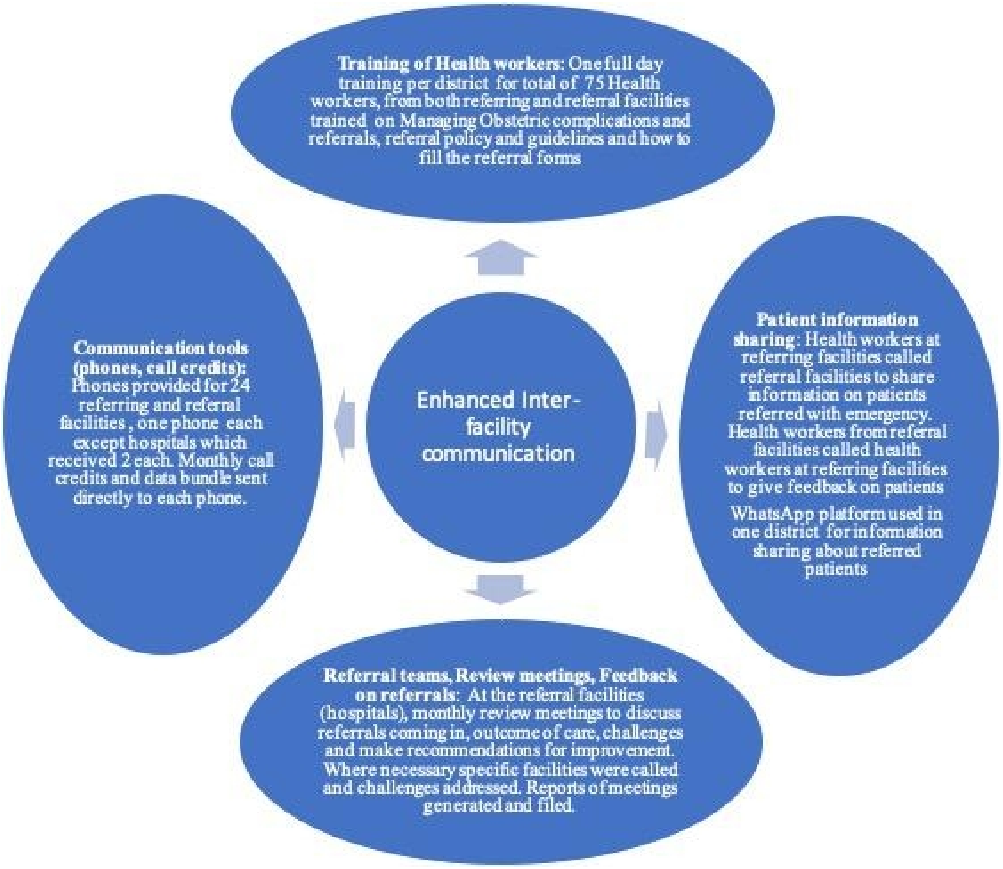

Diagrammatic representation of detailed intervention roll-out

198x171mm (72 x 72 DPI)

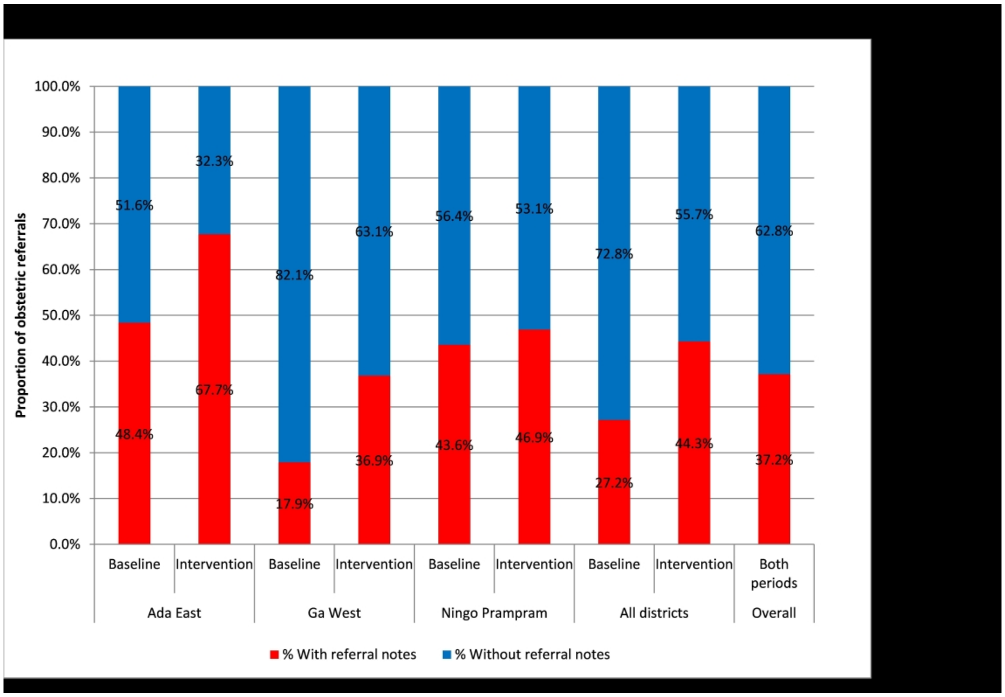

A Graph showing proportion of obstetric referrals with referral notes from the three districts, comparing baseline and intervention periods.

1428x982mm (96 x 96 DPI)

Standards for Reporting Qualitative Research (SRQR)*

http://www.equator-network.org/reporting-guidelines/srqr/

Title and abstract

	Page/line no(s).
**Title** - Concise description of the nature and topic of the study Identifying the study as qualitative or indicating the approach (e.g., ethnography, grounded theory) or data collection methods (e.g., interview, focus group) is recommended	2-4
**Abstract** - Summary of key elements of the study using the abstract format of the intended publication; typically includes background, purpose, methods, results, and conclusions	27-54

Introduction

**Problem formulation** - Description and significance of the problem/phenomenon studied; review of relevant theory and empirical work; problem statement	97-105
**Purpose or research question** - Purpose of the study and specific objectives or questions	105-107

Methods

**Qualitative approach and research paradigm** - Qualitative approach (e.g., ethnography, grounded theory, case study, phenomenology, narrative research) and guiding theory if appropriate; identifying the research paradigm (e.g., postpositivist, constructivist/ interpretivist) is also recommended; rationale**	112-113; 154-168
**Researcher characteristics and reflexivity** - Researchers' characteristics that may influence the research, including personal attributes, qualifications/experience, relationship with participants, assumptions, and/or presuppositions; potential or actual interaction between researchers' characteristics and the research questions, approach, methods, results, and/or transferability	169
**Context** - Setting/site and salient contextual factors; rationale**	111-113
**Sampling strategy** - How and why research participants, documents, or events were selected; criteria for deciding when no further sampling was necessary (e.g., sampling saturation); rationale**	Not Applicable
**Ethical issues pertaining to human subjects** - Documentation of approval by an appropriate ethics review board and participant consent, or explanation for lack thereof; other confidentiality and data security issues	213-220
**Data collection methods** - Types of data collected; details of data collection procedures including (as appropriate) start and stop dates of data collection and analysis, iterative process, triangulation of sources/methods, and modification of procedures in response to evolving study findings; rationale**	145-170

For peer review only - http://bmjopen.bmj.com/site/about/guidelines.xhtml

**Data collection instruments and technologies** - Description of instruments (e.g., interview guides, questionnaires) and devices (e.g., audio recorders) used for data collection; if/how the instrument(s) changed over the course of the study	145-170
**Units of study** - Number and relevant characteristics of participants, documents, or events included in the study; level of participation (could be reported in results)	245-250
**Data processing** - Methods for processing data prior to and during analysis, including transcription, data entry, data management and security, verification of data integrity, data coding, and anonymization/de-identification of excerpts	195-211; 122-125
**Data analysis** - Process by which inferences, themes, etc., were identified and developed, including the researchers involved in data analysis; usually references a specific paradigm or approach; rationale**	195-211
**Techniques to enhance trustworthiness** - Techniques to enhance trustworthiness and credibility of data analysis (e.g., member checking, audit trail, triangulation); rationale**	169-171; 210-211

Results/findings

**Synthesis and interpretation** - Main findings (e.g., interpretations, inferences, and themes); might include development of a theory or model, or integration with prior research or theory	241-356
**Links to empirical data** - Evidence (e.g., quotes, field notes, text excerpts, photographs) to substantiate analytic findings	270-274; 282-284; 325-327; 335-339

Discussion

**Integration with prior work, implications, transferability, and contribution(s) to the field -** Short summary of main findings; explanation of how findings and conclusions connect to, support, elaborate on, or challenge conclusions of earlier scholarship; discussion of scope of application/generalizability; identification of unique contribution(s) to scholarship in a discipline or field	359-367; 376-484
**Limitations** - Trustworthiness and limitations of findings	372-375

Other

**Conflicts of interest** - Potential sources of influence or perceived influence on study conduct and conclusions; how these were managed	498
**Funding** - Sources of funding and other support; role of funders in data collection, interpretation, and reporting	496

*The authors created the SRQR by searching the literature to identify guidelines, reporting standards, and critical appraisal criteria for qualitative research; reviewing the reference lists of retrieved sources; and contacting experts to gain feedback. The SRQR aims to improve the transparency of all aspects of qualitative research by providing clear standards for reporting qualitative research.

For peer review only

**The rationale should briefly discuss the justification for choosing that theory, approach, method, or technique rather than other options available, the assumptions and limitations implicit in those choices, and how those choices influence study conclusions and transferability. As appropriate, the rationale for several items might be discussed together.

Reference:

O'Brien BC, Harris IB, Beckman TJ, Reed DA, Cook DA. **Standards for reporting qualitative research: a synthesis of recommendations.** *Academic Medicine*, Vol. 89, No. 9 / Sept 2014 DOI: 10.1097/ACM.0000000000000388

For peer review only

STROBE Statement—checklist of items that should be included in reports of observational studies

	Item No.	Recommendation	Page No.	Relevant text from manuscript
**Title and abstract**	1	(*a*) Indicate the study's design with a commonly used term in the title or the abstract	2-4	
		(*b*) Provide in the abstract an informative and balanced summary of what was done and what was found	27-54	
**Introduction**				
Background/rationale	2	Explain the scientific background and rationale for the investigation being reported	70-105	
Objectives	3	State specific objectives, including any prespecified hypotheses	105-107	
**Methods**				
Study design	4	Present key elements of study design early in the paper	109-116	
Setting	5	Describe the setting, locations, and relevant dates, including periods of recruitment, exposure, follow-up, and data collection	119-131; 105- 112; 132-143	
Participants	6	(*a*) *Cohort study*—Give the eligibility criteria, and the sources and methods of selection of participants. Describe methods of follow-up<br>*Case-control study*—Give the eligibility criteria, and the sources and methods of case ascertainment and control selection. Give the rationale for the choice of cases and controls<br>*Cross-sectional study*—Give the eligibility criteria, and the sources and methods of selection of participants	146-151	
		(*b*) *Cohort study*—For matched studies, give matching criteria and number of exposed and unexposed<br>*Case-control study*—For matched studies, give matching criteria and the number of controls per case	Not Applicable	
Variables	7	Clearly define all outcomes, exposures, predictors, potential confounders, and effect modifiers. Give diagnostic criteria, if applicable	132-144; 145-153	
Data sources/ measurement	8*	For each variable of interest, give sources of data and details of methods of assessment (measurement). Describe comparability of assessment methods if there is more than one group	146-153	
Bias	9	Describe any efforts to address potential sources of bias	Not applicable	
Study size	10	Explain how the study size was arrived at	Study aimed at all referrals to hospital	

Continued on next page

For peer review only

For peer review only - http://bmjopen.bmj.com/site/about/guidelines.xhtml

Quantitative variables	11	Explain how quantitative variables were handled in the analyses. If applicable, describe which groupings were chosen and why	197-205
Statistical methods	12	(*a*) Describe all statistical methods, including those used to control for confounding	197-205
		(*b*) Describe any methods used to examine subgroups and interactions	Not applicable
		(*c*) Explain how missing data were addressed	Focus of work involved missing data
		(*d*) *Cohort study*—If applicable, explain how loss to follow-up was addressed<br>*Case-control study*—If applicable, explain how matching of cases and controls was addressed<br>*Cross-sectional study*—If applicable, describe analytical methods taking account of sampling strategy	Not applicable
		(*e*) Describe any sensitivity analyses	None
**Results**			
Participants	13*	(a) Report numbers of individuals at each stage of study—eg numbers potentially eligible, examined for eligibility, confirmed eligible, included in the study, completing follow-up, and analysed	241-250
		(b) Give reasons for non-participation at each stage	Not applicable
		(c) Consider use of a flow diagram	Not applicable
Descriptive data	14*	(a) Give characteristics of study participants (eg demographic, clinical, social) and information on exposures and potential confounders	Focus on referral notes not on participants
		(b) Indicate number of participants with missing data for each variable of interest	Focus of work involved missing data
		(c) *Cohort study*—Summarise follow-up time (eg, average and total amount)	Not applicable
Outcome data	15*	*Cohort study*—Report numbers of outcome events or summary measures over time	241 -250
		Case-control study—Report numbers in each exposure category, or summary measures of exposure	
		Cross-sectional study—Report numbers of outcome events or summary measures	
Main results	16	(*a*) Give unadjusted estimates and, if applicable, confounder-adjusted estimates and their precision (eg, 95% confidence interval). Make clear which confounders were adjusted for and why they were included	241-256; 287-315
		(*b*) Report category boundaries when continuous variables were categorized	None
		(*c*) If relevant, consider translating estimates of relative risk into absolute risk for a meaningful time period	None

Continued on next page

For peer review only - http://bmjopen.bmj.com/site/about/guidelines.xhtml

Other analyses	17	Report other analyses done—eg analyses of subgroups and interactions, and sensitivity analyses	None
**Discussion**			
Key results	18	Summarise key results with reference to study objectives	359-367
Limitations	19	Discuss limitations of the study, taking into account sources of potential bias or imprecision. Discuss both direction and magnitude of any potential bias	372-380
Interpretation	20	Give a cautious overall interpretation of results considering objectives, limitations, multiplicity of analyses, results from similar studies, and other relevant evidence	382-484
Generalisability	21	Discuss the generalisability (external validity) of the study results	478-484; 465-468
**Other information**			
Funding	22	Give the source of funding and the role of the funders for the present study and, if applicable, for the original study on which the present article is based	496

*Give information separately for cases and controls in case-control studies and, if applicable, for exposed and unexposed groups in cohort and cross-sectional studies.

Note: An Explanation and Elaboration article discusses each checklist item and gives methodological background and published examples of transparent reporting. The STROBE checklist is best used in conjunction with this article (freely available on the Web sites of PLoS Medicine at http://www.plosmedicine.org/, Annals of Internal Medicine at http://www.annals.org/, and Epidemiology at http://www.epidem.com/). Information on the STROBE Initiative is available at www.strobe-statement.org.

For peer review only

For peer review only - http://bmjopen.bmj.com/site/about/guidelines.xhtml
